# Mapping of sex hormones in the testicular vein of healthy men

**DOI:** 10.1210/clinem/dgag070

**Published:** 2026-04-01

**Authors:** Antonia Hufnagel, Hanne Frederiksen, Jakob Albrethsen, Trine Holm Johannsen, Anders Juul, Ruben Juhl Jensen, Anders Rehfeld, Romain Barrès

**Affiliations:** The Novo Nordisk Foundation Centre for Basic Metabolic Research, Faculty of Health and Medical Sciences, University of Copenhagen, 2200 Copenhagen, Denmark; Department of Growth and Reproduction, Copenhagen University Hospital—Rigshospitalet, 2100 Copenhagen, Denmark; International Center for Research and Research Training in Endocrine Disruption of Male Reproduction and Child Health (EDMaRC), Copenhagen University Hospital—Rigshospitalet, 2100 Copenhagen, Denmark; Department of Growth and Reproduction, Copenhagen University Hospital—Rigshospitalet, 2100 Copenhagen, Denmark; International Center for Research and Research Training in Endocrine Disruption of Male Reproduction and Child Health (EDMaRC), Copenhagen University Hospital—Rigshospitalet, 2100 Copenhagen, Denmark; Department of Growth and Reproduction, Copenhagen University Hospital—Rigshospitalet, 2100 Copenhagen, Denmark; International Center for Research and Research Training in Endocrine Disruption of Male Reproduction and Child Health (EDMaRC), Copenhagen University Hospital—Rigshospitalet, 2100 Copenhagen, Denmark; Department of Clinical Biochemistry, Copenhagen University Hospital—Rigshospitalet, Copenhagen, Denmark; Department of Growth and Reproduction, Copenhagen University Hospital—Rigshospitalet, 2100 Copenhagen, Denmark; International Center for Research and Research Training in Endocrine Disruption of Male Reproduction and Child Health (EDMaRC), Copenhagen University Hospital—Rigshospitalet, 2100 Copenhagen, Denmark; Department of Clinical Medicine, University of Copenhagen, 2200 Copenhagen, Denmark; Department of Diagnostic Radiology, Copenhagen University Hospital—Rigshospitalet, 2100 Copenhagen, Denmark; Department of Growth and Reproduction, Copenhagen University Hospital—Rigshospitalet, 2100 Copenhagen, Denmark; International Center for Research and Research Training in Endocrine Disruption of Male Reproduction and Child Health (EDMaRC), Copenhagen University Hospital—Rigshospitalet, 2100 Copenhagen, Denmark; Department of Clinical Biochemistry, Copenhagen University Hospital—Rigshospitalet, Copenhagen, Denmark; Department of Clinical Biochemistry, North Zealand Hospital, 3400 Hillerød, Denmark; Department of Cellular and Molecular Medicine, Faculty of Health Sciences, University of Copenhagen, 2200 Copenhagen, Denmark; The Novo Nordisk Foundation Centre for Basic Metabolic Research, Faculty of Health and Medical Sciences, University of Copenhagen, 2200 Copenhagen, Denmark; Institut de Pharmacologie Moléculaire et Cellulaire, Université Côte D’Azur, CNRS and Inserm, 06560 Valbonne, France

**Keywords:** reproductive hormones, steroidogenesis, male reproduction, testicular vein, reference ranges

## Abstract

**Context:**

The testis produces hormones controlling sex differentiation, development, and reproduction. Their quantification in physiological conditions can aid our understanding of the endocrine function of the testis.

**Objective:**

To measure the concentrations of steroid hormones and FSH, LH, inhibin B, anti-Müllerian hormone (AMH), and insulin-like factor 3 (INSL3) in the testicular and femoral veins, femoral artery, and seminal fluid in healthy men.

**Design:**

Sample collection was made by angiographic catheterization under fluoroscopy from the testicular and femoral veins. Femoral artery samples were collected via needle puncture and seminal fluid was isolated from semen samples. Hormones were measured via liquid chromatography mass spectrometry and immunoassays.

**Participants:**

Seven volunteers (aged 22-49 years) without self-reported diseases and normal semen quality were included.

**Main outcomes:**

Concentrations of steroid hormones, FSH, LH, inhibin B, AMH, and INSL3 in the testicular vein, femoral artery, femoral vein, and seminal fluid.

**Results:**

Expectedly, testosterone was 66-fold higher in the testicular vein compared to the femoral artery, but surprisingly, levels of 11-deoxycortisol and 11-deoxycorticosterone were 4- and 20-fold higher in the testicular vein compared to the femoral artery. In line with their production site in the testis, INSL3 and inhibin B were 200- and 2-fold higher in the testicular vein compared to the femoral artery. Unexpectedly, concentration of AMH was not different between the testicular vein and femoral artery despite its documented production in the testis.

**Conclusion and relevance:**

Our results provide insight into the endocrine function of the testis under physiological conditions and constitute a reference for further research in male reproductive function.

The testis secretes a variety of hormones such as testosterone, inhibin B, anti-Müllerian hormone (AMH), and insulin-like factor 3 (INSL3). Measurements of testicular hormones can be useful to the clinic, notably for the diagnosis of disorders of sex development, infertility, testicular dysfunction, or malignancies.

The most well-known testicular hormone is testosterone, vital for, eg, male sex development and spermatogenesis, and regulated by the pituitary hormones LH and FSH via the hypothalamic-pituitary-testicular axis. Testosterone is used as a biomarker for multiple male reproductive disorders, such as primary hypogonadism (1). Other androgens, such as DHT, are also equally crucial for sex development, and measurements of DHT can, eg, be used to diagnose 5-alpha reductase deficiency, a rare condition characterized by underdeveloped male genitalia (2). Other hormones in the steroidogenesis pathways like estrogens are connected to male reproductive function via their influence on sperm production (3). Besides steroid hormones, the testis also secretes the peptide hormones AMH, involved in male development in fetal life (4), INSL3, controlling testicular descend in the fetus (5), and inhibin B, which regulates FSH secretion and is an important marker for spermatogenesis (6). Deficiencies or excess production of all these hormones can thereby lead to diseases associated with reduced fertility and incomplete sex differentiation. Additionally, abnormally high levels of testicular hormones can be indicative of some types of testicular tumors or hyperplasias (eg, some Leydig cell tumors) (7).

Characterizing the endocrine function of the testis is therefore a valuable resource to understand testicular dysfunction, explore new biomarkers for testicular disease, and better understand male reproduction. This can be achieved in vivo by employing samples directly from the testicular vein, which is used in rare cases in clinical practice (eg, to detect unilateral Leydig cell hyperplasia (8, 9), to identify testicular tumors (7), to understand rare diseases such as androgen insensitivity syndrome) (10). Most research studies assessing the biosynthesis site of the testicular hormones using testicular vein samples collected these during varicocele or, less often, hernia surgeries, or from infertile men in connection with testicular biopsies (11-13). These studies have shown that concentrations of testosterone, estradiol, inhibin B, and INSL3 are elevated in the testicular vein compared with concentrations in peripheral blood (11-13), whereas concentrations of AMH were surprisingly found to be similar in the testicular vein and in the periphery despite the notion that AMH is exclusively produced in the testis (12). These results suggest that there could be more to unravel regarding testicular hormone production and raise the question whether these measures were affected by varicocele, hernia, or infertility in the studied participants (14). Additionally, the previous studies only measured selected testicular hormones and not all 19 metabolites of the steroidogenesis pathway. There is thereby a need to investigate testicular vein hormone levels in healthy men and to employ novel state-of-the-art liquid chromatography tandem mass spectrometry (LC-MS/MS) techniques to include measurements of all relevant steroid hormones.

Here, we investigated a large panel of hormones to assess all 19 metabolites of the steroidogenesis pathway including precursors of androgens, gluco- and mineralocorticoids, and estrogens. We mapped the entire steroidogenesis pathway via modern LC-MS/MS methods and measured peptide hormones produced in the testis (AMH, inhibin B, INSL3), nontesticular hormones such as the pituitary hormones FSH and LH, and the sex hormone-binding globulin (SHBG). These play a role in male reproductive function by regulating hormone secretion in the testis (LH and FSH) or regulating the bioavailability of sex steroids such as testosterone, DHT, and estradiol (SHBG).

To comprehensively study the endocrine function of the testis, we measured hormones involved in male reproduction not only in the testicular vein but also in the femoral artery and vein to get insight into the changes in hormone levels as blood is circulating through the testis. Our measures allow to assess whether hormones are indeed secreted by the testis and to evaluate nonspecific artery-vein differences in the periphery. Seminal fluid samples were also analyzed to further map secretion dynamics and mode of secretion of testicular hormones, as different male hormones are found in the seminal fluid in varying degrees (15) and as some testicular hormones have been described to be secreted luminally into the seminiferous tubules (16).

## Methods

### Sample collection

**Figure 1:**
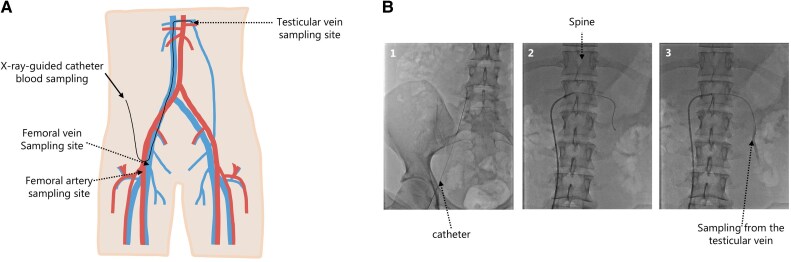
**Locations of blood sampling.** (A) Blood was sampled from the femoral and testicular vein and femoral artery (image drawn in Adobe Fresco). (B) A catheter was inserted into the femoral vein (1) and guided to the testicular vein (2), where a contrast agent was injected to ensure correct placement of the catheter before a blood sample was drawn (3).

Samples were collected from seven healthy men recruited for this study. Inclusion criteria were: healthy males; 18 to 50 years old; body mass index between 18.5 and 25; and normal sperm quality according to the World Health Organization (17) (>40% motility and >15 million sperm cell/mL). Exclusion criteria were: known disease; anemia; use of pharmaceuticals; use of illicit drugs; and smoking. The study was approved by the Ethical Committee of the Capital Region of Denmark (H-19064878) and was performed based on the principles of the Declaration of Helsinki and Good Clinical Practice guidelines. All participants were overnight fasted before sample collection in the morning. First, an ejaculated semen sample was provided in sterile plastic containers (Sarstedt collection cups) after at least 48 hours of abstinence. For the blood sampling, local anesthesia was injected into the skin of the right groin, in which the femoral vein was punctured, an introducer placed, and a blood sample taken from the femoral vein. All samples were taken in the morning. Conventional angiographic catheterization during fluoroscopy was used to locate and sample selectively from the testicular and femoral vein (Fig. 1). The last sample was taken from needle puncture in the right femoral artery, which was manually compressed for 10 minutes after sampling. After 1 hour of rest, participants were free to go home. For all blood samples, the first drawn blood (dead volume of the tubing, 3-5 mL) was drawn into a syringe before the real sample was collected and was thereafter reinfused into the participant to minimize the volume of the blood taken. For the hormone analysis, 2 × 3.5 mL were collected from all blood sampling sites in CAT serum Separator Clot Activator tubes (3.5 mL Vacuette), gently inverted 5 times and stored at room temperature (RT) until further processed.

### Sample processing

Semen samples were liquefied at RT for 15 to 30 minutes. Raw semen quality, including sperm motility and concentration, were measured using the Lensehooke semen quality device (LenseHooke X1 Pro with Lensehooke CS1 Semen Test Cassette; data in Table S1 (18)). Samples were then centrifuged (1000*g*, 10 minutes, RT) and seminal fluid taken off to be spun 1 additional time (3000*g*, 10 minutes, RT) to obtain pure seminal fluid that was then aliquoted and frozen at −80 °C for storage. Blood samples were left at RT for at least 30 minutes (processing times for each sample can be found in Table S2 (18)). Samples were then centrifuged (3000 rpm, 10 minutes, 5 °C) and aliquoted into separate vials for steroid hormone and nonsteroid hormone and SHBG analysis for storage at −80 °C.

### Hormone analysis

Serum concentrations of 19 steroid metabolites, including progestogens, androgens, estrogens, and mineralo- and glucocorticoids were determined using in-house isotope-dilution online-TurboFlow LC-MS/MS methods as previously reported in detail for androgens, corticosteroids, and estrogens (19, 20). For measurement of estrogens, isotope diluted serum samples were purified by liquid-liquid extraction before loading on the LC-MS/MS system, which for both methods was equipped with a heated electrospray ionization source running in both positive and negative mode and with aqueous ammonium fluoride and methanol as mobile phases on the analytical column. Samples were analyzed in batches together with ordinary patient samples for, respectively, androgens/corticosteroids and estrogens. Besides samples, each batch included blanks (water), calibration, and control material, consisting of 3 controls (low, mid, high concentration) in triplicates. The inter-assay coefficients of variations (CVs) were within the individual accepted limits for all metabolites. Seminal fluid samples were analyzed for the concentrations of steroid metabolites using the same LC-MS/MS methods as for steroid metabolites in serum (19, 20). Serum INSL3 was determined by LC-MS/MS as previously reported, with a CV below 15% (21, 22).

Serum concentrations of LH and FSH were measured by chemiluminescence immunoassays (Siemens Atellica IM 1300 Analyzer, RRID:SCR_027876) with CVs of 30% and 20% (CV of 7% for both hormones for samples with high concentration), respectively (23). Serum concentrations of SHBG and AMH were determined by chemiluminescence immunoassays (Access 2, Beckman Coulter, RRID:SCR_019607) with CVs of 10% and 7%, respectively (24). Additionally, the serum concentration of AMH was also determined by a sandwich electrochemiluminescence immunoassay (Roche Cobas 8000 e 801 analyzer, RRID:SCR_025257).

The serum concentration of inhibin B was determined by an enzyme-linked immunosorbent assay (inhibin B GenII ELISA, Beckman-Coulter, Brea, CA, USA, RRID:AB_2827405) with a CV of 10% (25).

Limits of detection (LODs) of all measured hormones can be found in Table 1 (26-39).

**Table 1:** Measurements of progestogens, androgens, estrogens, gluco- and mineralocorticoids, peptide hormones and sex-hormone binding protein in the femoral artery and vein, testicular vein and seminal fluid of the 7 participants (LOD, half-life and mean ± SD shown)

Hormone	LOD	Half-life	Femoral vein (mean ± SD, n = 7)	Femoral artery (mean ± SD, n = 7)	Testicular vein (mean ± SD, n = 7)	Seminal fluid (mean ± SD, n = 6)
**Progestogens, androgens, and estrogens**						
Progesterone (nM)	0.036	5 minutes (26)	0.42 ± 0.14	0.49 ± 0.18	20.49 ± 10.38	0.15 ± 0.01 (n = 1 <LOD)
17-Hydroxypregnenolone (nM)	0.193	—	11.11 ± 5.77	22.8 ± 16.77	361.14 ± 124.81	2.13 (n = 5 <LOD)
17-Hydroxyprogesterone (nM)	0.033	47 minutes (27)	4.23 ± 2.07	4.25 ± 1.46	422.34 ± 185.38	0.54 ± 0.16
DHEA (nM)	4.4	1-3 hours (28)	31.8 ± 15.9	38.7 ± 23.9	218.9 ± 94.0	<LOD
DHEAS (nM)	4.514	10-20 hours (28)	6898 ± 2871	6729 ± 2751	6859 ± 2671	2314 ± 1073
Androstenedione (nM)	0.031	77 minutes (27)	2.98 ± 0.89	3.46 ± 1.10	40.19 ± 24.58	0.59 ± 0.26
Testosterone (nM)	0.031	10 minutes (29)	20.3 ± 6.3	17.8 ± 6.0	1350.1 ± 710.4	0.6 ± 0.3
Dihydrotestosterone (nM)	0.118	—	1.45 ± 0.58	1.43 ± 0.57	12.29 ± 5.74	1.02 ± 0.80
Estrone (pM)	2.93	20-30 minutes (30)	113.6 ± 27.5	71.4 ± 14.1	272.5 ± 85.5	25.6 ± 13.3
Estrone sulfate (nM)	0.025	10-12 hours (30)	1.66 ± 0.42	1.74 ± 0.57	1.82 ± 0.64	1.25 ± 0.69
Estradiol (pM)	4.04	20-30 minutes (30)	91.1 ± 26.2	54.2 ± 12.2	2607.6 ± 894.6	22.6 ± 16.4
Estriol (pM)	12.3	3-60 minutes (31)	<LOD	<LOD	<LOD	<LOD
**Gluco- and mineralocorticoids**						
11-Deoxycorticosterone (nM)	0.029	—	0.12 ± 0.04	0.15 ± 0.09	2.46 ± 0.89	Below LOD
Corticosterone (nM)	0.217	55 minutes (32)	10.9 ± 6.3	29.1 ± 31.8	17.5 ± 12.1	1.8 ± 0.6
Aldosterone (nM)	0.038	20-30 minutes (33)	0.27 ± 0.12	0.20 ± 0.21	0.16 ± 0.06	<LOD
11-Deoxycortisol (nM)	0.042	4.7 minutes (34)	0.67 ± 0.33	0.85 ± 0.46	2.73 ± 1.08	0.20 ± 0.08 (n = 5 <LOD)
Cortisol (nM)	5.565	66 minutes (35)	337 ± 96	403 ± 153	343 ± 124	33 ± 9
Cortisone (nM)	0.642	—	63.2 ± 9.5	60.1 ± 11.0	71.9 ± 13.8	24.8 ± 5.2
21-Deoxycortisol (nM)	0.035	—	<LOD	<LOD	<LOD	<LOD
**Other reproductive hormones**						
FSH (IU/L)	0.3	3-4 hours (36)	3.58 ± 1.35	3.66 ± 1.47	3.54 ± 1.35	n.a.
LH (IU/L)	0.07	20 minutes (36)	3.18 ± 1.91	3.33 ± 1.86	3.09 ± 1.84	n.a.
SHBG (nM)	0.33	7 days (37)	38.04 ± 13.60	36.93 ± 13.39	37.82 ± 15.07	n.a.
Inhibin B (ng/L)	3	1.5–3 hours (38)	203 ± 44	196 ± 45	493 ± 155	2403 ± 3652 ng/L
INSL3 (ug/L)	0.01	—	0.69 ± 0.32	0.63 ± 0.30	144.18 ± 97.56	<LOD
AMH (pM)	0.14	27 hours (39)	43.60 ± 9.68	42.81 ± 9.55	43.87 ± 10.02	65.85 ± 108 pM

For the seminal fluid samples, measurements were only done for 6 participants because of limited sample volume. The literature can give slightly different half-life measurements dependent on multiple factors (disease, age, sex) so the values shown here give an estimate of the range. For some hormones, no well-supported measurement could be found and therefore no value is shown.

Abbreviations: AMH, anti-Müllerian hormone; DHEA(S), dehydroepiandrosterone (sulfate); INSL3, Insulin-like peptide 3; LOD, limit of detection; n.a., not analyzed.

All hormone analyses were performed at the Department of Growth and Reproduction, Copenhagen University Hospital—Rigshospitalet, except for the Cobas 8000 AMH measurement, which was performed at the Department of Clinical Biochemistry, Copenhagen University Hospital—Rigshospitalet. The hormone and analytical laboratories were accredited for medical examination according to the standard DS/EN ISO 15189 by the Danish Accreditation Fund.

### Data analysis

Statistical analysis of hormone data across the different sampling locations and different participants was performed using R Studio with a linear mixed effects model (lmer). The participant was included as a random effect with the location of sampling (testicular vein, femoral artery, femoral vein) as a categorical variable: lmer (measurement ∼ location + (1|participant|). Where assumptions for normality of residuals and homogeneity were not met, the data were log transformed. Seminal fluid concentrations were not included in the statistical model because of the different type of matrix making direct comparisons difficult but are shown in the graphs for visualization and to provide a comprehensive mapping of the hormones in all relevant matrices. Hormone levels in the seminal fluid could only be measured for 6 of the 7 participants because of limited material obtained. Visualization was done using ggplot; all raw data (Table S3 (18)) are available in the supplement.

## Results

### Progestogens, androgens, and estrogens

**Figure 2:**
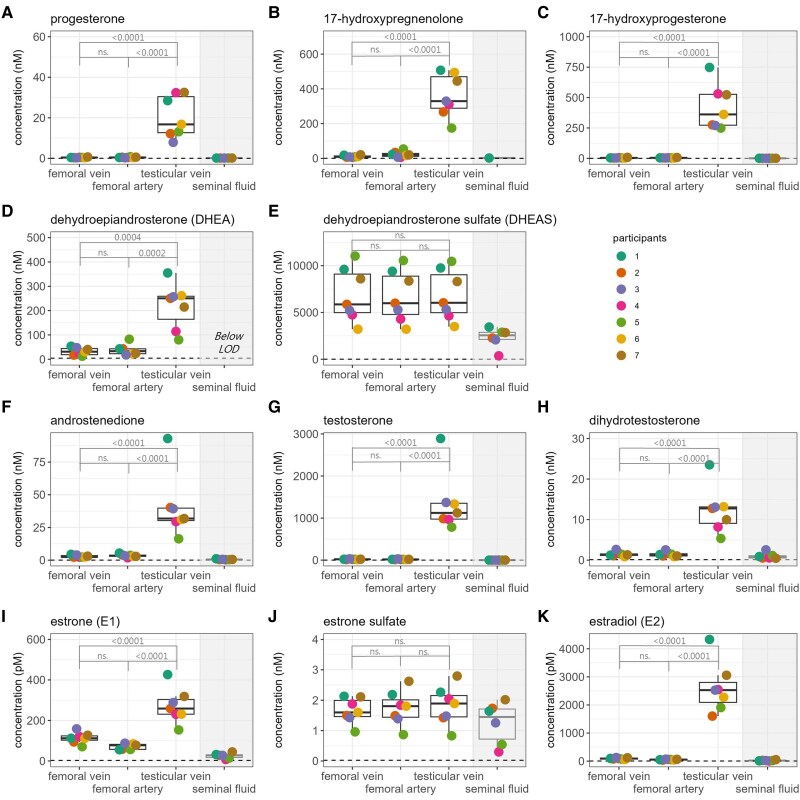
**Progestogens, androgens, and estrogens.** Measurements of (A) progesterone, (B) 17-hydroxypregnenolone, (C) 17-hydroxyprogesterone, (D) dehydroepiandrosterone (DHEA), (E) dehydroepiandrosterone sulfate (DHEAS), (F) androstenedione, (G) testosterone, (H) dihydrotestosterone, (I) estrone (E1), (J) estrone sulfate, (K), and estradiol (E2) across the different compartments. Results of the lmer analysis are shown (seminal fluid is not included in the statistical analysis), LODs are indicated by the dashed line.

The concentrations of most progestogens and androgens (17-hydroxypregnenolone, 17-hydroxyprogesterone, progesterone, dehydroepiandrosterone [DHEA], androstenedione, testosterone, and DHT) showed no differences between femoral artery and vein but were significantly higher in samples from the testicular vein, compared to femoral vein or artery samples (Fig. 2, Table 1). Similarly, estrone and estradiol were the same in the femoral artery and vein but significantly higher concentrations were observed in the testicular vein compared to the other blood compartments (Fig. 2, Table 1). There were no significant differences between all blood compartments for dehydroepiandrosterone sulfate (DHEAS) and estrone sulfate (Fig. 2, Table 1).

Overall, seminal fluid concentrations of the progestogens, androgens, and estrogens were lower than those found in blood, with DHEA and 17-hydroxypregnenolone levels below the LODs (Fig. 2, Table 1). Concentrations of progesterone were also very low in the seminal fluid with 1 measurement being below LOD (Fig. 2, Table 1). Seminal fluid concentrations of DHEAS, estrone, and estradiol were below all corresponding blood concentrations, and DHEAS levels correlated positively with the levels of the hormone in the blood. Estrone sulfate, however, was measured at similar concentrations as in blood. None of these hormones showed significant correlation between concentrations in semen and semen volume.

### Mineralo- and glucocorticoids

**Figure 3:**
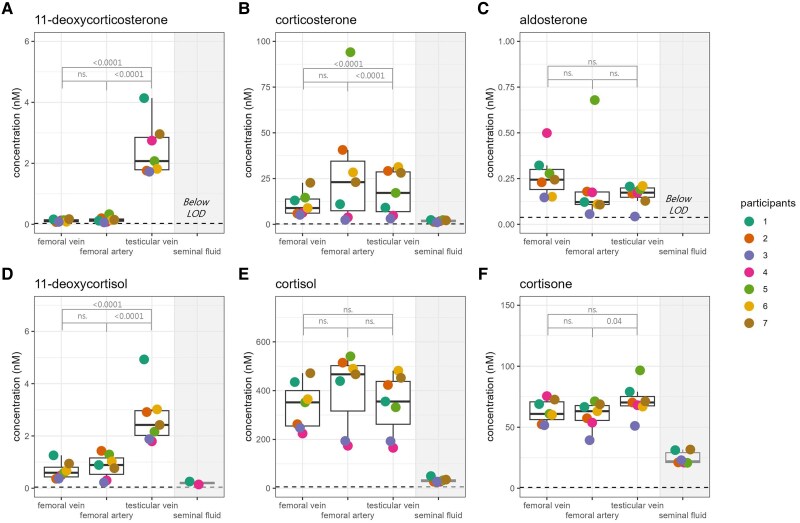
**Mineralo- and glucocorticoids.** Measurements of (A) 11-deoxycorticosterone, (B) corticosterone, (C) aldosterone, (D) 11-deoxycortisol, (E) cortisol, and (F) cortisone across the different compartments. Results of the lmer analysis are shown (seminal fluid is not included in the statistical analysis); LODs are indicated by the dashed line.

The concentrations of the glucocorticoids cortisone and 11-deoxycortisol and the mineralocorticoid 11-deoxycorticosterone were similar between femoral artery and vein but were significantly higher in samples from the testicular vein, compared to the femoral artery and vein (Fig. 3, Table 1).

There were no significant differences between the blood compartments for cortisol, corticosterone, and aldosterone (Fig. 3, Table 1). As expected, 21-deoxycortisol could not be detected in any blood samples. Corticosterone, cortisol, and cortisone were detectable in the seminal fluid samples below the levels in the blood samples (Fig. 3, Table 1) and were not correlating with semen volume. 11-deoxycortisol was only detectable in seminal fluid in 2 of the 6 samples.

### Other reproductive hormones

**Figure 4:**
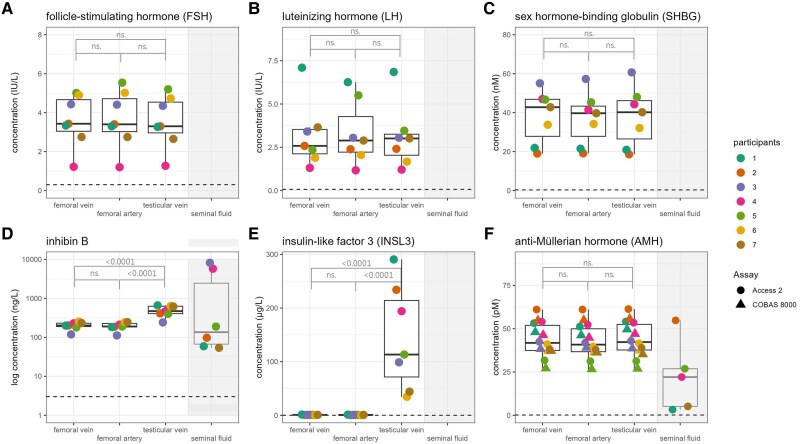
**Other reproductive hormones.** Measurements of (A) FSH, (B) LH, (C) SHBG, (D) inhibin B, (E) insulin-like factor 3 (INSL3), and (F) anti-Müllerian hormone (AMH) (2 independent measurements shown, statistical analysis performed on the 2 measurements together) across the different compartments. Results of the lmer analysis are shown (seminal fluid is not included in the statistical analysis); LODs are indicated by the dashed line.

The concentrations of inhibin B and INSL3 were similar between femoral artery and vein but were significantly higher in samples from the testicular vein, compared to the other blood samples, whereas FSH, LH, SHBG, and AMH were not different between the 3 blood compartments (Fig. 4A-4E). Because of the unexpected finding that AMH is not significantly increased in the testicular vein despite its described production in the testis, AMH was measured in 2 independent laboratories, with both measurements shown in Fig. 4F. Seminal fluid concentrations were only measured for inhibin B and AMH because of technical restrictions. Concentrations of inhibin B and AMH were quite variable in the seminal fluid and did not show correlations with the hormone levels in the different blood compartments or with semen volume.

## Discussion

**Figure 5:**
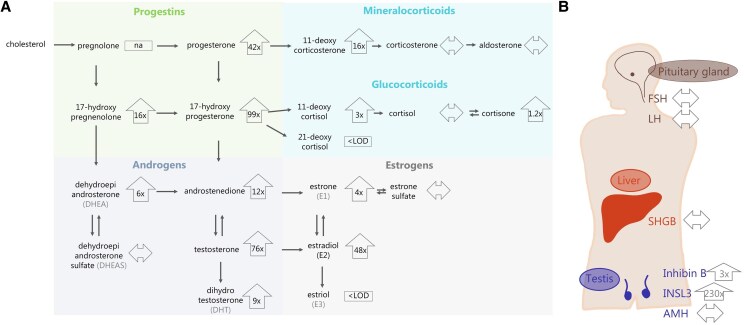
**Summary of the presented data.** (A) Nineteen different steroid hormones were measured in this study—their biogenesis and grouping into progestogens, androgens, estrogens, mineralo- and glucocorticoids is shown here. (B) Additionally, 5 peptide hormones and the hormone-binding protein SHBG produced by the pituitary gland, liver, and testis were measured (image drawn in Adobe Fresco). Fold change between testicular vein and femoral artery is shown in arrows. Unchanged levels between these 2 compartments are indicated with a horizontal arrow. n.a.: not analyzed.

Our study provides new knowledge into the endocrine function of the testis through the comprehensive measurement of 19 steroids of the steroidogenesis pathway and well-known peptide hormones and SHBG in the testicular vein, femoral artery and vein, and seminal fluid of healthy men (Fig. 5).

Concentrations of the Leydig cell marker testosterone, its precursors and INSL3, were significantly higher in the testicular vein compared to the femoral artery in accordance with previous studies of varicocele patients (11, 14, 40). DHEA is primarily synthesized in the adrenal gland but a minor part of circulating DHEA is produced in the testis (41, 42). We found much higher testicular production of DHEA than anticipated, whereas DHEAS concentrations were the same in all blood compartments. These results make biological sense as sulfation of DHEA takes place exclusively in the adrenal gland. Given the high level of DHEA we measured in testicular vein samples, our data indicate that more DHEA may be produced in the testis than previously thought. A similar observation was made for DHT in accordance with the fact that 30% of the circulating DHT is converted from testosterone directly in the testis (43).

Interestingly, some of the progestogens and androgens, such as 17-hydroxypregnenolone, progesterone, and DHEA, could not be detected in seminal fluid or were present at very low levels. In a previous study, high concentrations of DHEA were detected in seminal fluid (44). 17-hydroxypregnenolone and progesterone have also previously been detected in seminal fluid using LC-MS/MS methods (15), both of which were below or close to the LODs in our samples.

Estrone and estradiol were detected at higher concentrations in the testicular vein compared to the other blood compartments. This is in line with data from 1972 in which hernia surgery patients showed increased estradiol levels in the testicular vein (45), showing that the human testis secretes estrogens. Estrone sulfate has been thought to be secreted from the testis but this hypothesis was not confirmed in an analysis of testicular vein samples from varicocele patients (46). In our dataset, we similarly observed equal concentrations of estrone sulfate across the different blood compartments. Estrone sulfate, however, was detected in the seminal fluid samples at similar concentrations to the blood samples. This is different to most other measured hormones, where seminal fluid levels were lower than in the blood samples. Higher levels of estrone sulfate in the seminal fluid compared to serum samples have been reported previously in normospermic individuals (47). Collectively, this could indicate active transport of some of the testicular hormones into the seminal fluid, as the relative levels of these hormones differ between these matrices, which would not be expected if diffusion alone was responsible.

Concentrations of 11-deoxycortisol and 11-deoxycorticosterone, initially thought to be exclusively synthesized by the adrenal gland, were higher in the testicular vein compared to the femoral artery in our data, indicating testicular production. In rat studies, 11-deoxycorticosterone and 11-deoxycortisol similarly appear to be synthesized in the testis (48). Our data also show that cortisone levels are moderately higher in the testicular vein compared to the femoral artery, which has not been reported before. Cortisol, on the other hand, is exclusively synthesized by the adrenal gland (49), as confirmed by our data. It has been shown by others that cortisol levels in seminal fluid correlate with levels in the blood circulation, indicating that it enters the testis from the blood circulation to reach the seminal fluid (44) and/or reach the ejaculate via other routes.

Circulating inhibin B consists of 2 subunits (the α and βB subunits), which are both produced in the Sertoli cells of the prepubertal testis (50), whereas the βB subunit appears to be produced by spermatocytes and spermatids after puberty (51). Accordingly, we found elevated inhibin B concentrations in the testicular vein. The high INSL3 concentrations in testicular vein is expected due to its production by Leydig cells in the testis (52). The striking absence of increased levels of AMH in the testicular vein compared to blood samples from a peripheral vein has previously been reported in varicocele patients, questioning whether this was also the case in healthy men (12).

Our data indicate that AMH levels are indeed not higher in the testicular vein compared to the femoral artery and vein of healthy men. As it is well-established that AMH is secreted by Sertoli cells, this raises the question why it is not detected at higher concentrations in the testicular vein compared to the femoral artery and vein. In addition to varicocele, Goulis et al (12) mention several potential reasons for the similar AMH concentrations in testicular vein and periphery, including that a hitherto unidentified extratesticular synthesis site exists; this, however, is not supported by the fact that AMH is undetectable in anorchid boys (53-55). Alternatively, Sertoli cells secrete AMH across the apical domain after puberty directly into the lumen of the seminiferous tubules rather than to the basolateral interstitium, from where it would reach circulation (12). In our study, seminal fluid concentrations of AMH varied markedly across participants and showed no correlation with concentrations in blood. High variability in seminal fluid concentrations of AMH has been shown before (56). Despite such variability, the observed high or equal concentration in the seminal fluid compared to the blood compartment in our dataset could support the hypothesis that the apical mode of secretion is a reason for the unexpected absence of elevated AMH concentrations in the testicular vein. Another putative explanation could be the dynamics of AMH secretion. AMH secretion shows little or no circadian changes, whereas inhibin B—another product of the Sertoli cells—displays a clear circadian pattern of secretion (57). Additionally, studies in females have shown that the half-life of AMH is 27 hours (39). As reported in Table 1, this is clearly longer than the half-life of all other hormones except for SHBG. The long half-life combined with a constant secretion pattern could lead to an equilibrium of AMH in the blood circulation, potentially explaining the almost equal concentrations in the different blood compartments. Finally, that AMH is secreted in 2 forms (inactive pro-AMH and active AMH), which are measured together using routine assays, could potentially also play a part in the observed similar AMH concentrations across blood compartments (58). Data from mice experiments with recombinant human AMH suggest that pro-AMH is only cleaved to AMH in the gonads and not in the circulation (59). However, in our study, we did not measure pro-AMH and AMH separately, whereby no clear conclusion can be drawn regarding this aspect for humans. These AMH findings merit further investigations of pro-AMH and AMH separately and of the secretory dynamics of AMH in the circulation.

Our detailed quantification of hormones in the testicular vein of healthy men provides unprecedented insight into the endocrine function of the testis under physiological conditions. Although some measurements align with previous findings from varicocele, hernia, and infertile patients, others represent novel observations; most notably, the substantial concentrations of 11-deoxycortisol and 11-deoxycorticosterone in the testicular vein, suggesting their secretion by the testis. Taken together, our findings advance understanding of male reproductive hormone secretion and regulation in normal physiology and establish a reference for future clinical research.

## Data Availability

Original data generated and analyzed during this study are included in this published article in the Supplement (18).

## Abbreviations

AMH: anti-Müllerian hormone
CV: coefficient of variation
DHEA: dehydroepiandrosterone
DHEA(S): dehydroepiandrosterone (sulfate)
INSL3: Insulin-like peptide 3
LC-MS/MS: liquid chromatography tandem mass spectrometry
LOD: limit of detection
RT: room temperature
